# Profiling of Long Non-coding RNAs and mRNAs by RNA-Sequencing in the Hippocampi of Adult Mice Following Propofol Sedation

**DOI:** 10.3389/fnmol.2018.00091

**Published:** 2018-03-23

**Authors:** Jun Fan, Quan Zhou, Yan Li, Xiuling Song, Jijie Hu, Zaisheng Qin, Jing Tang, Tao Tao

**Affiliations:** ^1^Department of Anesthesiology, Nanfang Hospital, Southern Medical University, Guangzhou, China; ^2^Department of Neurobiology, School of Basic Medical Sciences, Southern Medical University, Guangzhou, China; ^3^Department of Orthopedics and Traumatology, Nanfang Hospital, Southern Medical University, Guangzhou, China; ^4^Department of Anesthesiology, Affiliated Hospital of Guangdong Medical University, Zhanjiang, Guangdong, China

**Keywords:** propofol, long non-coding RNA, hippocampus, RNA-sequencing, neurotoxicity

## Abstract

Propofol is a frequently used intravenous anesthetic agent. The impairment caused by propofol on the neural system, especially the hippocampus, has been widely reported. However, the molecular mechanism underlying the effects of propofol on learning and memory functions in the hippocampus is still unclear. In the present study we performed lncRNA and mRNA analysis in the hippocampi of adult mice, after propofol sedation, through RNA-Sequencing (RNA-Seq). A total of 146 differentially expressed lncRNAs and 1103 mRNAs were identified. Bioinformatics analysis, including gene ontology (GO) analysis, pathway analysis and network analysis, were done for the identified dysregulated genes. Pathway analysis indicated that the FoxO signaling pathway played an important role in the effects of propofol on the hippocampus. Finally, four lncRNAs and three proteins were selected from the FoxO-related network for further validation. The up-regulation of lncE230001N04Rik and the down-regulation of lncRP23-430H21.1 and lncB230206L02Rik showed the same fold change tendencies but changes in Gm26532 were not statistically significant in the RNA-Seq results, following propofol sedation. The FoxO pathway-related proteins, PI3K and AKT, are up-regulated in propofol-exposed group. FoxO3a is down-regulated at both mRNA and protein levels. Our study reveals that propofol sedation can influence the expression of lncRNAs and mRNAs in the hippocampus, and bioinformatics analysis have identified key biological processes and pathways associated with propofol sedation. Cumulatively, our results provide a framework for further study on the role of lncRNAs in propofol-induced or -related neurotoxicity, particularly with regards to hippocampus-related dysfunction.

## Introduction

Propofol, the most widely used intravenous anesthetic agent in medical practice (Moseley et al., 1988), is noted among its analogs for its shorter onset and better recovery quality. Over the past few decades, several researchers have reported its protective effects in ischemic-reperfusion injury, including cerebral ischemic injury. Most of these reports attributed propofol's neuroprotective effect to its anti-inflammatory (Nie et al., 2015; Samir et al., 2015) and antioxidant (Ucar et al., 2015; Hsiao et al., 2016) properties. However, in the clinical setting, propofol is often administered to patients with an intact central nervous system (CNS). Thus, it is especially important to investigate the effects of propofol on an intact CNS.

In the last decade, there is increasing evidence toward propofol's neurotoxic effect. Literatures describe multiple mechanisms, including the influence on dendritic development (Vutskits et al., 2005), induced neuronal apoptosis (Yan et al., 2017), increased cell death in neurons and oligodendrocytes (Krzisch et al., 2013), impaired maturation of neurons in newborns (Krzisch et al., 2013), and disturbance of the differentiation of neurons and astrocytes (Erasso et al., 2013). More importantly, some of these reports suggest that propofol's neurotoxicity impairs hippocampus-related learning and memory functions (Vutskits et al., 2005; Erasso et al., 2013; Krzisch et al., 2013; Liu et al., 2017; Qiao et al., 2017; Yan et al., 2017). Hippocampus-related learning and memory may be regulated by different processes, such as neurogenesis in the developmental and adult stage, long-term potential and synaptic plasticity. A recently reported study suggested that propofol induces an increase in TNF-α, short- or long-term neuronal apoptosis, neuronal loss, and synaptic loss (Chen et al., 2016). Another study on the same group of participants indicated that TNF-α contributes to propofol-induced neuronal apoptosis via the PI3K/AKT signaling pathway (Deng et al., 2017). In addition, it has been shown that maternal exposure to propofol during the late stages of pregnancy can impair learning and memory in the newborn by inactivating the BDNF-TrkB signaling pathway in the hippocampus of the fetus (Zhong et al., 2016). In our previous study (Fan et al., 2016), we demonstrated that propofol possesses the ability to influence the expression of several microRNAs in neural stem cells (NSCs). Other studies have also shown that propofol inhibits NSC neurogenesis through a mechanism involving the miR-141-3p/IGF2BP2 axis (Jiang et al., 2017). However, the role of long non-coding RNA (lncRNA) in propofol-induced neurotoxicity, in the hippocampus, is still unknown.

LncRNA is defined as longer than 200 nucleotides in length and possessed almost no coding potential (Rinn and Chang, 2012). LncRNAs can regulate gene expression in four steps: epigenetic regulation, transcriptional regulation, post-transcriptional regulation, and translational regulation (Sun et al., 2017). Recently, several reports have provided new insights into the mechanism by which lncRNAs may regulate gene expression. Specific mechanisms include that of scaffolding and recruiting multiple regulatory proteins, genetic imprinting and chromatin remodeling, producing microRNA sponges, shaping, and utilizing three-dimensional nuclear structures and so on (Jandura and Krause, 2017; Sun et al., 2017; Bunch, 2018). Interestingly, in the brain, a large proportion of tissue-specific lncRNAs are preferentially expressed in specific regions or within different cell types (Derrien et al., 2012). These lncRNAs in the CNS participate in many aspects of brain function and in CNS development, from early neural differentiation to late-stage synaptogenesis (Briggs et al., 2015). Ramos et.al (Ramos et al., 2015) found that Pnky, a conserved lncRNA, may regulate neurogenesis in embryonic and postnatal NSC populations. Further, lncRNA-Map2k4 may regulate neuronal proliferation and apoptosis through a miR-199a/FGF1 pathway (Lv, 2017), and lncRNA Meg3 acts as a functional regulator in the regulation of the PTEN/PI3K/AKT signaling cascade, during the process of synaptic plasticity in neurons (Tan et al., 2017). Although several studies suggest that microRNA, another non-coding RNA, plays an important role in propofol-induced neurotoxicity, the relationship between propofol and an lncRNA has only been mentioned in one report, which indicates that propofol can inhibit lncRNA HOTAIR, which induces apoptosis in cervical cancer cells (Zhang et al., 2015). Therefore, the question remains whether propofol can affect lncRNA expression profiles in the hippocampus, and if so, how is propofol-induced lncRNA expression altered in propofol-induced neurotoxicity?

In the present study, we adopted a previously reported propofol-induced neurotoxicity mouse model (Krzisch et al., 2013) and evaluated the extent of impairment of learning and memory functions using the Morris water maze. Then the different expression patterns of lncRNAs and mRNAs in the hippocampi of propofol-sedated mice were identified by RNA-Seq. Through bioinformatics analysis of the genes with varying expression patterns, we discovered the potential biological processes and signaling pathways that may play important roles during the process of propofol sedation, and closely connect with the hippocampus-related learning and memory functions.

## Materials and methods

### Experimental animals

Eight to ten-week-old SPF (specific pathogen free) wild type C57BL6/J male mice (25–30 g) were purchased from the Laboratory Animal Center of Southern Medical University, Guangzhou, China. The mice were maintained under standard laboratory conditions [12-h light-dark cycles (lights on from 7:00 a.m. to 7:00 p.m.) with free access to food and water] unless otherwise indicated. All the protocols were approved by the Animal Use and Committee for Research and Education of Southern Medical University (Protocol number: SYXK-2015-0056). The experimental animals underwent adaptation to the environment in the course of a 2-week breeding period, and were subsequently subjected to experiments.

### Anesthesia procedure

**Figure 1:**
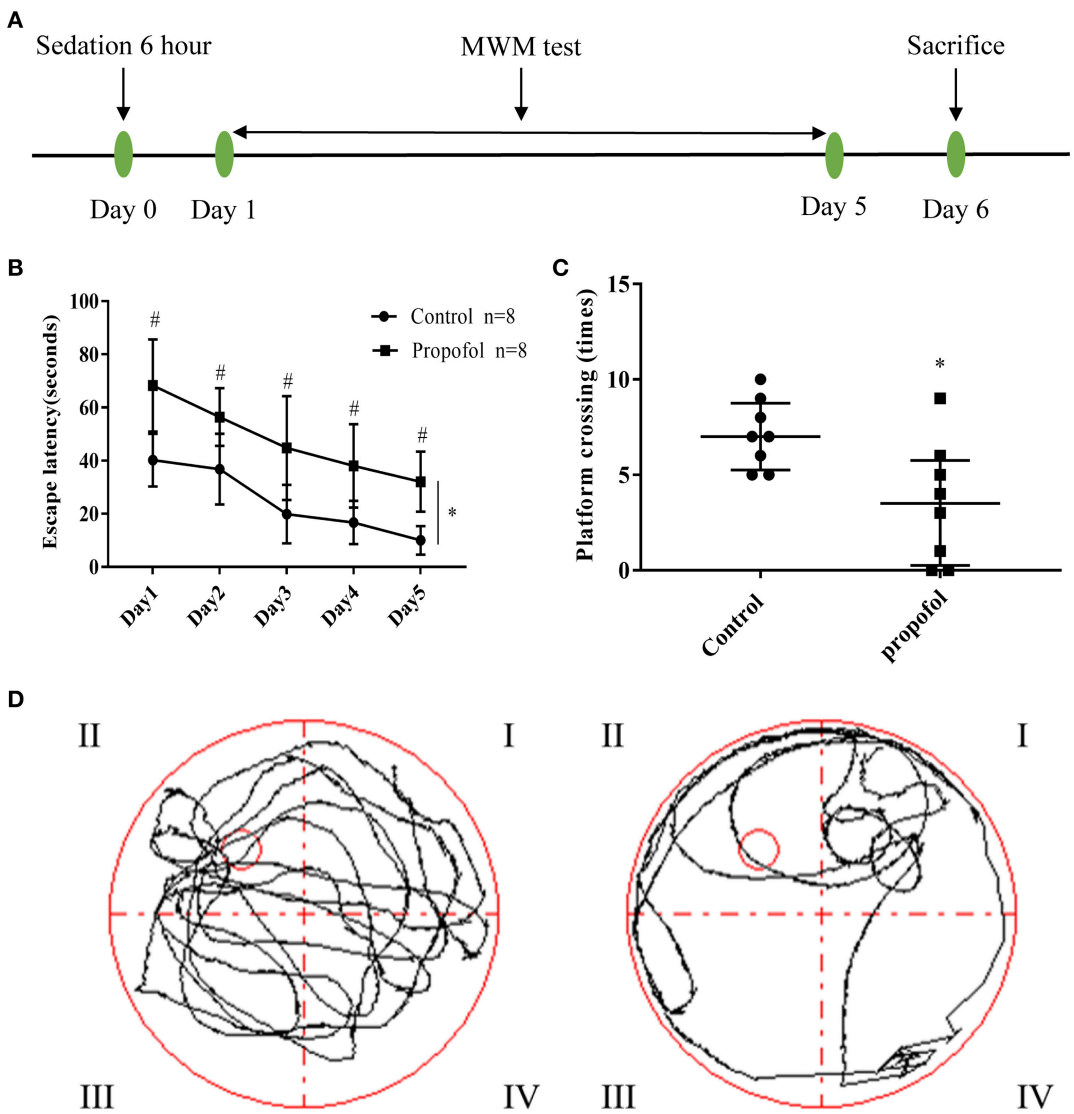
Experimental design and behavioral readouts of MWM tests. **(A)**. Schematic illustration of the experimental design; **(B)** The latency of mice to reach the platform, in the two groups. Two-way ANOVA showed that propofol-induced anesthesia increases the escape latency of MWM, when compared with the control group. (*P < 0.05), the Bonferroni test shows that the propofol-treated mice have a longer escape latency compared to that of the mice receiving control treatment, from day 1 to day 5 (^#^*P* < 0.05 vs. control group at each day). Results are presented as mean ± standard error (*n* = 8, ^*^P < 0.05); **(C)** Number of platform crossings in the probe trial. Results are presented as median and interquartile range (*n* = 8, ^*^P < 0.05); **(D)** Representative searching swimming paths of two mice in the probe tests. (Left: Control group, Right: Propofol group).

Adult mice were randomized into two groups (control group and propofol group; *n* = 8 in each group). The protocol used in this study was based on previous report (Krzisch et al., 2013). On day 0, mice in the Prop (propofol) group were anesthetized for 6 h; an initial intraperitoneal injection of propofol (100 mg/kg, propofol 1% medium-chain triglycerides, AstraZeneca, London, UK) was administered, followed by five subsequent injections at 50 mg/kg, at the rate of one injection per hour. The sedative effects were confirmed by noting the absence of the clip tail reflection and righting reflex. The Con (control) group was treated according to the same procedure, but instead using the same volumes of medium-chain and long-chain triglycerides (20%, Huarui, Wuxi, China). All mice were kept in a water bath (Yiheng, Shanghai, China) to maintain body temperature at 37°C during the entire period under anesthesia. Animals continued to feed till the next day, and were subjected to the behavioral test (Figure 1A).

### Morris water maze (MWM)

The Morris water maze test (*n* = 8 for each group) was performed to assess the spatial learning and memory functions (Figure 1A). The maze consists of a circular polypropylene pool (110 cm in diameter and 20 cm in height) that was filled with tap water, up to an approximate height of 15 cm, at room temperature (23±3°C). The water was made opaque using milk powder, to ensure camouflage of the escape platform. A white escape platform (4.5 cm in diameter, 14.5 cm in height) was submerged 1 cm below the water surface and placed at the midpoint of one quadrant (the target quadrant). A colorful flag was added for visibility of the platform trial. Three pre-set extra mazes were placed on the wall of the testing room and the swimming activities were recorded via a digital video camera above the pool, which were analyzed using the Digi Behave system. During the testing period, the room was dimly lit with diffuse white light.

The platform was positioned in the middle of the northwest (NW) quadrant for all mice. Each mouse was released from a predetermined, pseudo-random start location (south, east, west, or north) in the tank to receive daily training, for 4 consecutive days. Escape latency, in finding the submerged escape platform, was calculated for each trial. When the mouse failed to find the platform after 90 s, it was guided onto the platform and allowed to stay on it for ~10 s. After 4 days (day 1 to day 4) of training, mice were tested for spatial memory in a 90 s probe trial, with no platform present. The time spent in the target quadrant during the probe trial and the number of times the mice crossed the original location of the platform, were recorded. On the 5th day (day 5), mice were tested (four trials) using a visible platform that was placed in the same location within the northwest quadrant. Quadrant locations remained the same each time the mice were tested.

### Total RNA extraction

The day after the MWM test (day 6) (Figure 1A), three mice from each group were randomly selected and the right hippocampus of each mice was harvested for the next experiments. Each of the hippocampus samples were washed twice, in cold PBS (Hyclone, Logan, USA), and immediately stored at −80°C. Total RNA was isolated using a Trizol reagent (Invitrogen, Carlsbad, USA), according to the manufacturer's protocol. RNA degradation and contamination were monitored on 1% agarose gels and RNA purity was analyzed using the NanoPhotometer spectrophotometer (IMPLEN, CA, USA). RNA concentration was measured using the Qubit RNA Assay Kit in Qubit 2.0 Flurometer (Life Technologies, CA, USA). RNA Nano 6000 Assay Kit of the Bioanalyzer 2100 system (Agilent Technologies, CA, USA) was used to assess the integrity of the isolated RNA.

### Library preparation for lncRNA sequencing

A total of 3 μg of RNA, per sample, was used as input material for the RNA sample preparations. Firstly, ribosomal RNA was removed by the Epicentre Ribo-zero rRNA Removal Kit (Epicentre, Madison, USA), and the residual free rRNA was removed by ethanol precipitation. Subsequently, sequencing libraries were generated with the rRNA-depleted RNA using the NEBNext Ultra Directional RNA Library Prep Kit for Illumina (NEB, Ipswich, USA), according to the manufacturer's recommendations.

### Clustering and sequencing

The clustering of the index-coded samples was performed on a cBot Cluster Generation System using the TruSeq PE Cluster Kit v3-cBot-HS (Illumia, San Diego, USA), according to the manufacturer's instructions. Following cluster generation, the libraries were sequenced on an Illumina Hiseq 2000 platform and 100 bp paired-end reads were generated.

### Quality control

Clean data (clean reads) were obtained by removing reads containing adapter, reads containing ploy-N and low-quality reads from raw data. At the same time, Q20, Q30, and GC content of the clean data were calculated. The downstream analysis was done on high-quality clean data.

### Mapping to the reference genome

The reference genome and gene model annotation files were downloaded directly from a genome website (Reference genome: http://ftp.ensembl.org/pub/release-79/fasta/mus_musculus/dna/) (Annotation files: http://ftp.ensembl.org/pub/release-79/gtf/mus_musculus/). The index of the reference genome was built using Bowtie v2.0.6 and the paired-end clean reads were aligned with the reference genome using TopHat v2.0.9 (Trapnell et al., 2012).

### Transcriptome assembly

The mapped reads of each sample were assembled by both Scripture (beta2) (Guttman et al., 2010) and Cufflinks (v2.1.1) (Trapnell et al., 2012), following the reference. Both methods use spliced reads to determine the exon connectivity, via different approaches. Scripture uses a statistical segmentation model to distinguish expressed loci from experimental noise, and uses spliced reads to assemble the expressed segments. It reports all the statistically expressed isoforms in a given locus. On the other hand, Cufflinks uses a probabilistic model to simultaneously assemble and quantify the expression levels of a minimal set of isoforms, which provides a maximum-likelihood explanation to the expression data in a given locus (Cabili et al., 2011).

### Coding potential analysis

CNCI (Coding-Non-Coding-Index) (v2) (Sun et al., 2013); CPC (Coding Potential Calculator) (0.9-r2) (Kong et al., 2007); Pfam Scan (v1.3) (Punta et al., 2012) and PhyloCSF (phylogenetic codon substitution frequency) (v20121028) (Lin et al., 2011) were used to predict the coding potential of transcripts. Once the transcripts were predicted to have coding potential by either/all of the four tools listed above, they were filtered, and those without coding potential were included as our candidate set of lncRNAs.

### Target gene prediction

The cis and trans target mRNAs of the lncRNAs were used to predict their functions, due to a lack of adequate existing functional annotations of lncRNAs. A “cis role” indicates that the lncRNA acts on adjacent target genes. We searched the protein-coding genes 10k/100k upstream and downstream of the target lncRNA, and analyzed their functions. A “trans role” indicates that the lncRNA is used in identification, through the corresponding expression level. Although there were no more than 25 samples, we calculated the correlation of expression between lncRNAs and protein-coding genes with custom scripts; in addition, we clustered the genes from different samples with WGCNA (Langfelder and Horvath, 2008) to search common expression modules and then analyzed their function through functional enrichment analysis.

### Differential expression analysis

Cuffdiff (v2.1.1) was used to calculate fragments per kilobase million (FPKMs), of both lncRNAs and coding genes, in each sample. Gene FPKMs were computed by summation of the FPKMs of transcripts in each gene group. Cuffdiff provides statistical routines for determining differential expression in digital transcript or gene expression data, using a model based on the negative binomial distribution. For biological replicates, transcripts or genes with a *p*-value of < 0.05 were accepted as differentially expressed.

### GO and KEGG enrichment analysis

Gene Ontology (GO) enrichment analysis of differentially expressed genes, or lncRNA target genes, was implemented with DAVID (http://david.abcc.ncifcrf.gov/). GO terms with *p* < 0.05 were considered significantly enriched by differentially expressed genes. Kyoto Encyclopedia of Genes, and Genomes (KEGG) is a database resource for understanding high-level functions and effects of the biological system (http://www.genome.jp/kegg/). DAVID (http://david.abcc.ncifcrf.gov/) was also used to test the statistical enrichment of genes, or target genes of lncRNA, with differential expression in KEGG pathways. The networks of the pathways and pathway-related genes were constructed using Cytoscape (version 3.2.1) plugin ClueGO + Cluepedia application.

### Construction of the co-expression network

Significantly expressed mRNAs, which were involved in the FoxO signaling pathway, were superimposed onto the lncRNA-mRNA correlation network to determine their association with the lncRNAs. STRING (version 10.5) (Szklarczyk et al., 2017) was used to provide critical assessment and integration of protein-protein interactions. In the network, the circular nodes represent significantly expressed mRNAs and the diamond nodes represent the related lncRNAs; the black lines show connections between lncRNAs and their target mRNAs; the purple lines show integration of protein-protein interactions and the thicker lines represent a larger combined score.

### PCR and western blotting validation

As previously mentioned, total RNA was isolated. The first strand cDNA was generated using the Reverse Transcription System Kit (Takara, China), and real-time PCR was performed using SYBR Premix Ex Taq II kit (Takara, China) on an Applied Bio systems 7500 Real-time PCR System (ABI, USA), in triplicates. The expressions of lncRNA or mRNA were normalized by U6 and GAPDH, respectively. All the sequences of primers used are listed in Table S3. The fold change gene expression was calculated using the 2^−ΔΔCt^ method.

Western blot was performed, as previously described. The hippocampus protein samples (30 μg) were separated by SDS-polyacrylamide gels and then transferred to PVDF membranes. After blocking with 5% fat free milk, the membranes were incubated with PI3K (1:750 Wanleibio, China), AKT (1:750, Wanleibio, China), FoxO3a (1:600, Wanleibio, China) and GAPDH (1:5,000, Beijing Ray Antibody, China). Membranes were then incubated in the appropriate peroxidase-conjugated secondary antibodies (1:10,000, Beijing Ray Antibody, China), at room temperature. After washing, antibody-bound proteins were detected with the ImmobilonTM Western Chemiluminescent HRP Substrate Kit (Millipore, USA) and exposed to X-ray film (Kodak, USA) for 1–2 min. The results were normalized to GAPDH and quantified using Image J (version 1.6.0_24).

### Statistical analysis

In MWM tests, data in escape latency period was expressed as mean ± *SD*. The data for platform crossing times was not normally distributed and thus were expressed as median and interquartile range. There was no missing data for the variables of MWM, during the analysis. Interaction between time and group factors, in a two-way ANOVA with repeated measurements, was used to analyze the differences in learning curves (based on escape latency), between mice in the control group and mice treated with propofol in the MWM. Bonferroni analysis was used to compare the differences in escape latency between the control group and the propofol group, for each day. The Mann–Whitney test was used to determine the differences in platform crossing times, between the control and propofol conditions. An unpaired *t*-test was used to determine the differences in the levels of RNAs and proteins, between the control and propofol groups.

## Results

### Propofol exposure impaired spatial learning and memory in adult mice

The spatial learning and memory in adult mice were tested through MWM analysis, 1 day after propofol exposure. Two-way ANOVA showed that propofol anesthesia increased the escape latency of MWM when compared with the control group (Figure 1B) (*P* = 0.0002). The Bonferroni test revealed that the mice that received propofol had a longer escape latency, compared to the mice receiving the control treatment, from day 1 to day 5 (Figure 1B) (*P* day1 = 0.001, *P* day2 = 0.006, *P* day3 = 0.007, *P* day4 = 0.004, *P* day5 = 0.001). In the probe tests, the Mann–Whitney test showed that the number of platform crossings were significantly reduced in the propofol group (Figures 1C,D), (*P* = 0.0138). The data suggests that propofol exposure impairs spatial learning and memory in adult mice.

### RNA-seq analysis of hippocampus transcriptome-identified mRNAs and lncRNAs

**Figure 2:**
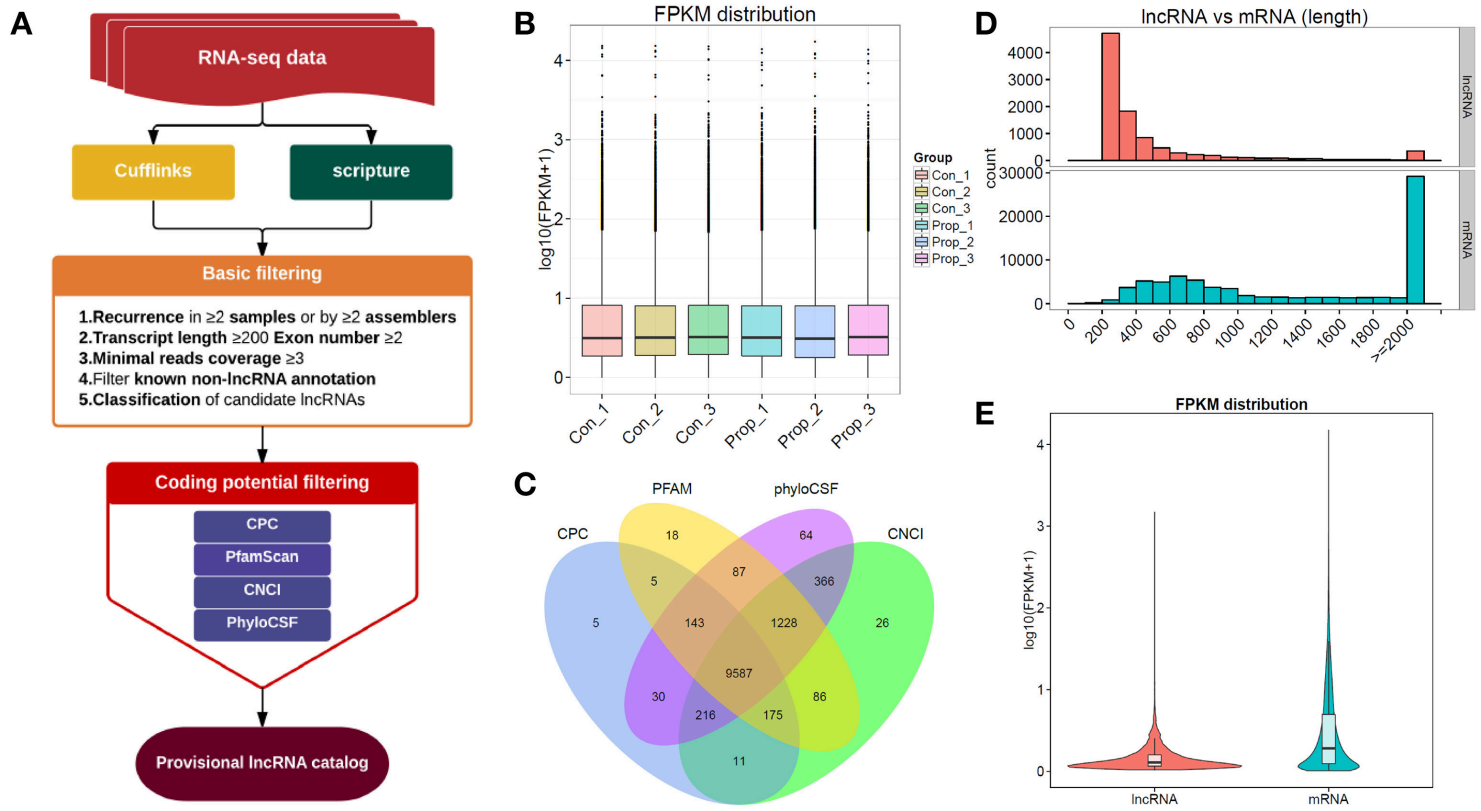
The comprehensive evaluation and screening of candidate lncRNAs. **(A)**. The main workflow for screening provisional lncRNAs; **(B)**. The box plots of FPKM distribution in different groups, showing no significant differences between the control and propofol groups; **(C)**. The Venn diagram of the coding potential of screened transcripts using CNCI, CPC, PFAM, and phyloCSF, 9587 transcripts were predicted to have no coding potential by all of the four methods; **(D)** The full length of mRNAs is longer than lncRNAs; **(E)** The violet plots of expression levels of lncRNAs and mRNAs.

Total RNA from the right hippocampi of all six treated mice was isolated and sequenced. A total of 384 million (Con group) and 417 million (Prop group) raw reads were obtained by RNA-Seq (Table S1). Approximately 365 million and 397 million clean reads from the Con and Prop groups were isolated, respectively, and almost 73.96% (Con group) and 70.74% (Prop group) of the clean reads were uniquely mapped to reference genome (Table S1), which were selected for subsequent experiments. The sequence reads were performed to map known gene types. Approximately 83.70% (Con group) and 82.54% (Prop group) of reads were mapped to coding genes, and around 1.50 and 1.42% of reads in the Con and Prop groups, respectively, were mapped to lncRNAs (Table S2). The expression level in each sample showed no differences in terms of FKPM values (Figure 2B).

### The results of screening provisional lncRNAs

The provisional lncRNAs were screened according to the workflow shown in Figure 2A. Five steps of basic filtering were applied: step 1—recurrence in ≥2 samples or by ≥2 assemblers; step2—transcript length ≥200bp, exon number ≥2; step3—minimal reads coverage ≥3; step4—filtration of known non-lncRNA annotation; step 5—classification of candidate lncRNAs. Four methods, CPC, CNCI, Pfam and PhyloCSF, were used to predict the coding potential. The transcript was eliminated if it was predicted to possess coding potential, by any or all of the four methods. The rest of the transcripts were selected as the candidate set of lncRNAs. We finally had 9587 transcripts that were predicted to have no coding potential, by all the four tools (Figure 2C), and all these transcripts were candidates for the following lncRNA research. There were also 80162 transcripts of mRNAs in total, with 77676 transcripts mapped to the reference genome, and the rest of them unknown novel mRNAs transcripts (data not shown).

### The comparison between mRNAs and unknown novel lncRNAs

To distinguish between the number of unknown lncRNAs and mRNAs in introns and exons, 9587 transcripts that were predicted to have no coding potential by all the four tools, were compared with mRNAs which can be mapped to the genome, which showed that mRNAs were of longer lengths (Figure 2D). On the other hand, lncRNAs presented with a lower expression level than mRNAs (Figure 2E). The results of such a comparison between mRNAs and unknown novel lncRNAs, supports the existing information on lncRNAs.

### Differentially expressed genes

**Figure 3:**
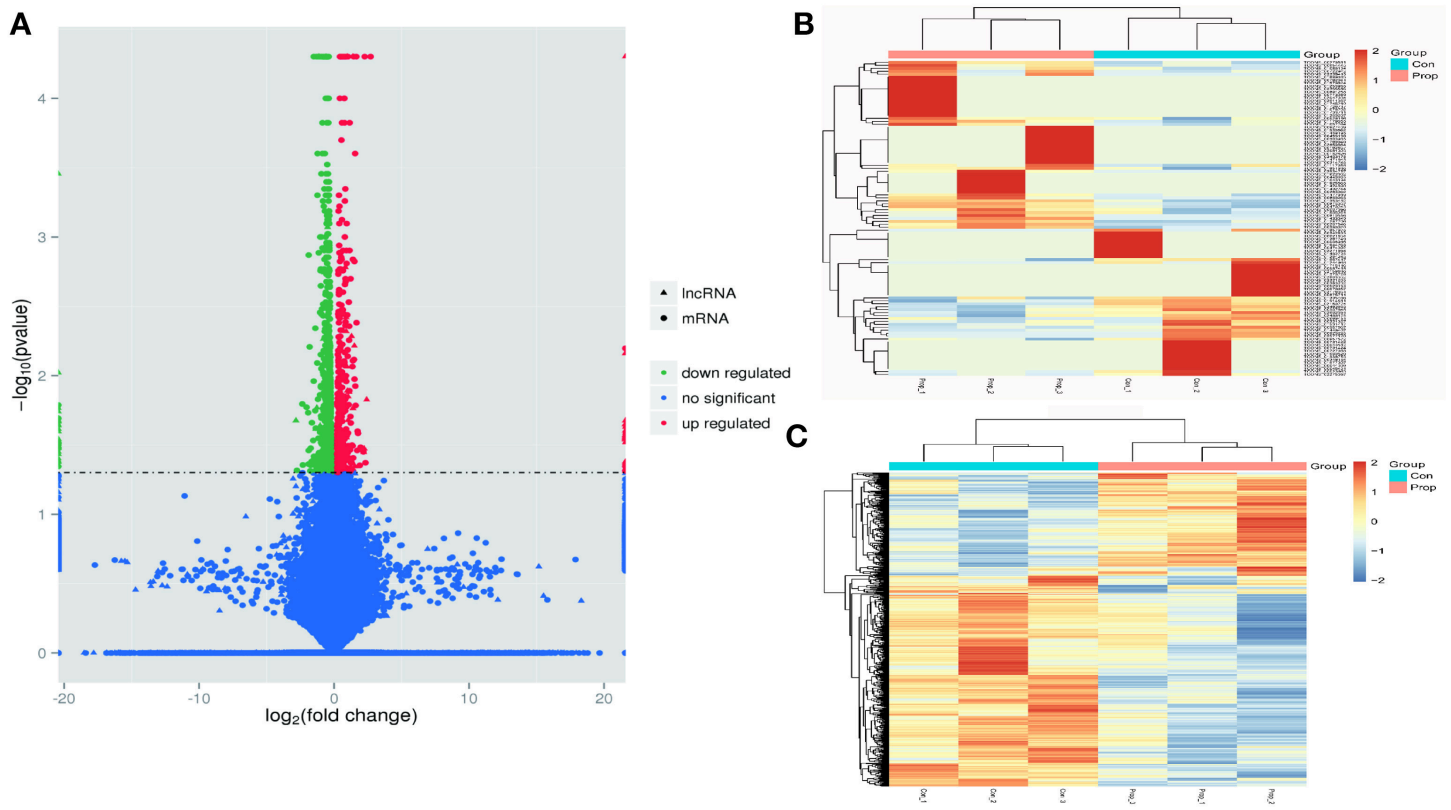
Transcriptome profile of RNA-Seq data distinguishing control and propofol groups. **(A)** A volcano plot of differentially expressed lncRNAs and mRNAs between control and propofol groups; **(B)** Unsupervised hierarchical clustering of the expression profiles of differentially expressed lncRNAs in the propofol group, compared with the control group; **(C)** Unsupervised hierarchical clustering of the expression profiles of differentially expressed mRNA in the propofol group compared with the control group.

Cuffdiff was used to detect differentially expressed genes between the Prop group and Con group, and 1249 differentially expressed transcripts were screened in total, including 433 up-regulated and 816 down-regulated ones in the Prop group (Figure 3A). Furthermore, there were 146 differentially expressed lncRNAs containing 77 up-regulated (20 known and 57 novel) and 69 down-regulated (19 known and 50 novel) genes, the heatmap of which is displayed in Figure 3B. In addition, 1103 mRNAs, containing 356 up-regulated (331 known and 25 novel mRNAs) and 747 down-regulated (678 known and 69 novel mRNAs) mRNAs, were also identified and are demonstrated as a heatmap (Figure 3C). Further, 6628 target genes (in 10k) and 15485 target genes (in 100k) in cis role, and 18834 target genes in trans role (data not shown) were predicted for further study.

### The GO and KEGG enrichment analysis of target genes

**Figure 4:**
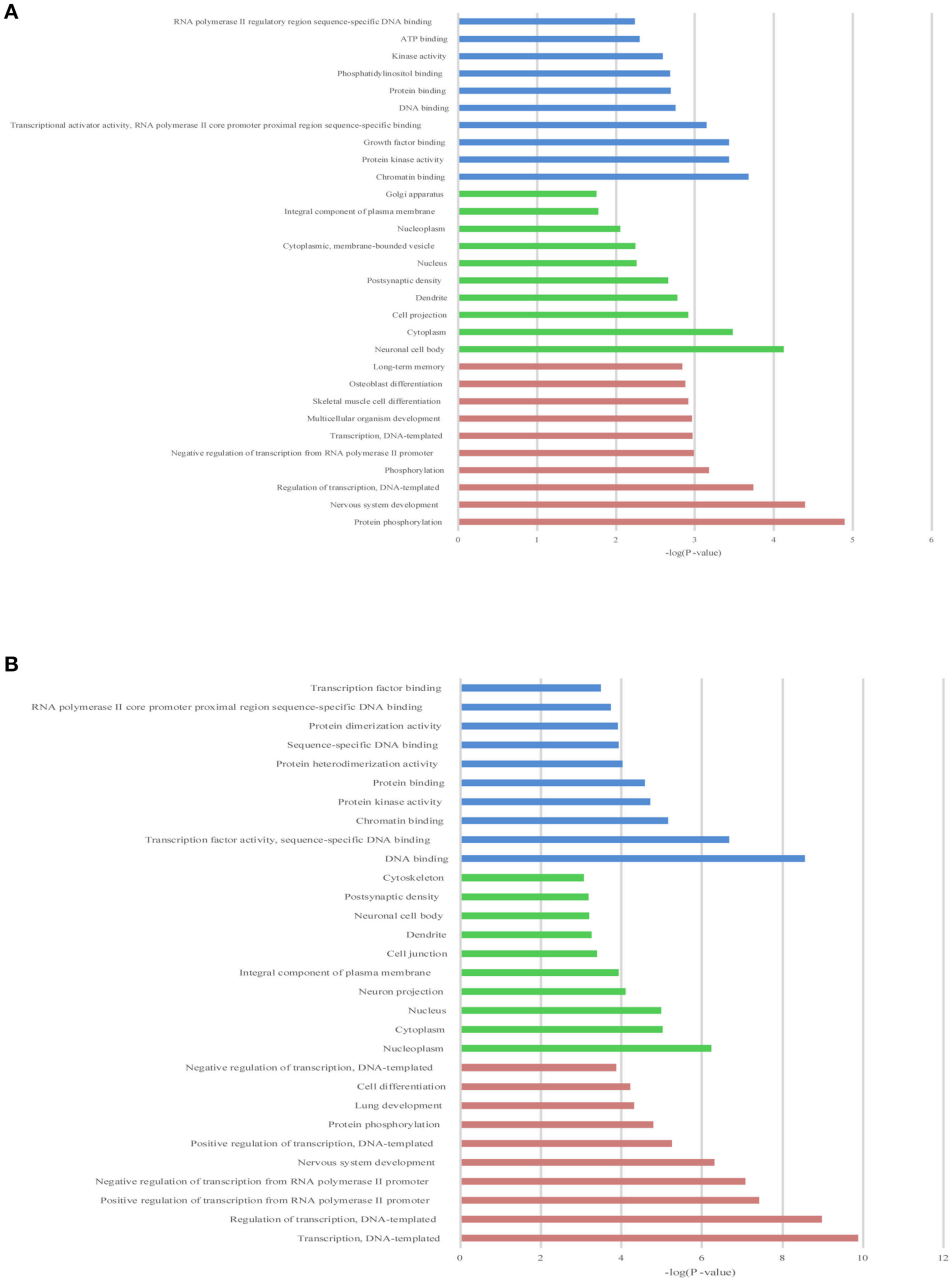
The top 10 enrichment scores in gene ontology (GO) enrichment analysis on target genes of differentially expressed lncRNAs. **(A)** Analysis of the up-regulated lncRNAs; **(B)** Analysis of the down-regulated lncRNAs. Red bars represent biological process terms; Green bars represent cell component terms; Blue bars represent molecular function terms.

**Figure 5:**
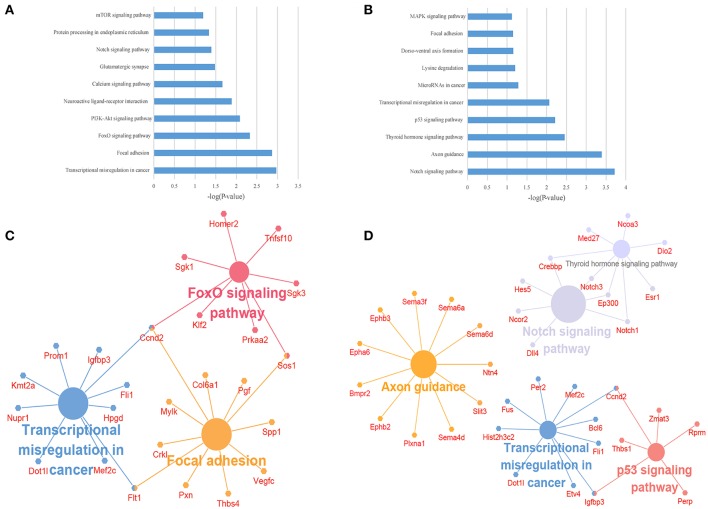
The KEGG pathway enrichment analysis on target genes of differentially expressed lncRNAs. **(A,B)** The top 10 enrichment scores in the KEGG pathway analysis of the target genes of the up-regulated **(A)** and down-regulated **(B)** lncRNAs; **(C,D)** The network of most enriched pathways of the up-regulated **(C)** and down-regulated **(D)** lncRNAs and related genes.

To illustrate the functions of the differentially expressed lncRNAs and their relationship with each other, GO and KEGG pathway enrichment analysis were conducted. In the GO analysis, we investigated the target genes of up-and down-regulated lncRNAs. All the results were ranked according to the enrichment score and the top 10 of each category are displayed in Figure 4. In the biological process analysis, 71 terms related to up-regulated lncRNAs were significantly enriched, while 90 processes in relation to down-regulated lncRNAs were significantly enriched. In the cellular component analysis, 12 terms associated with the up-regulated lncRNAs, and 26 terms linked to the down-regulated lncRNAs, were significantly enriched. In the molecular function analysis, 31 terms associated with the up-regulated lncRNAs, and 51 terms associated with the down-regulated lncRNAs, were significantly enriched. Results of the KEGG pathway analysis were also ranked according to the enrichment score, and the top 10 gene pathways associated with the target genes of up-regulated and down-regulated lncRNAs are listed in Figures 5A,B. Among these, MAPK and FoxO signaling pathways also appeared in the top 10 results of the mRNA KEGG analysis, indicating a close connection between these pathways and the effects of propofol. The network of the most enriched pathways and their related genes (Figures 5C,D) revealed that Ccnd2, Flt1, Crebbp, Notch3, Notch1, Ep300, and Igfbp3 were all cross-talk genes, which were associated with at least two pathways.

### The GO and KEGG enrichment of differentially expressed mRNAs

**Figure 6:**
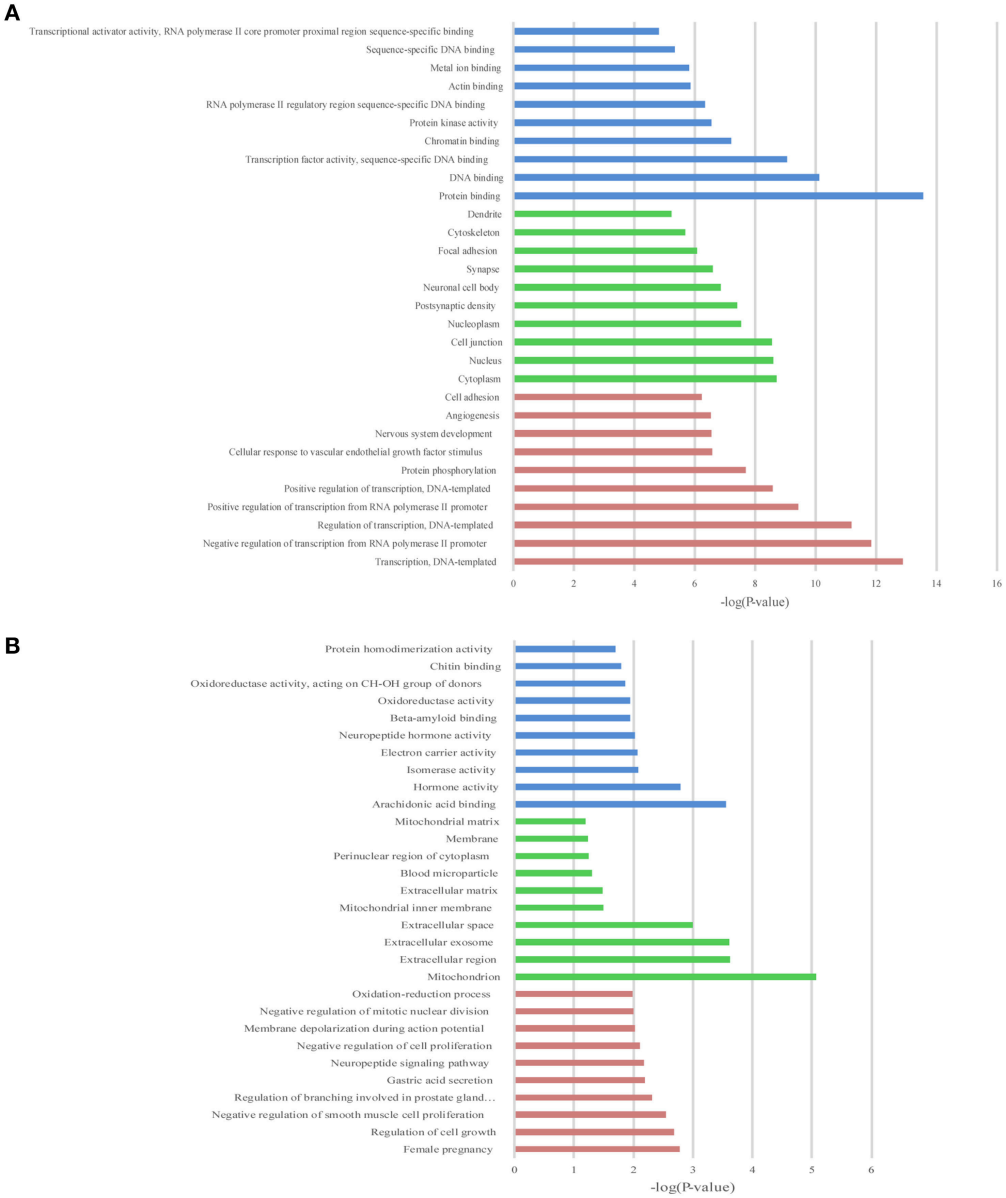
The top 10 enrichment scores in gene ontology (GO) enrichment analysis of differentially expressed mRNAs. **(A)** Analysis of the up-regulated mRNAs; **(B)** Analysis of the down-regulated mRNAs; Red bars represent biological process terms; Green bars represent cell component terms; Blue bars represent molecular function terms.

**Figure 7:**
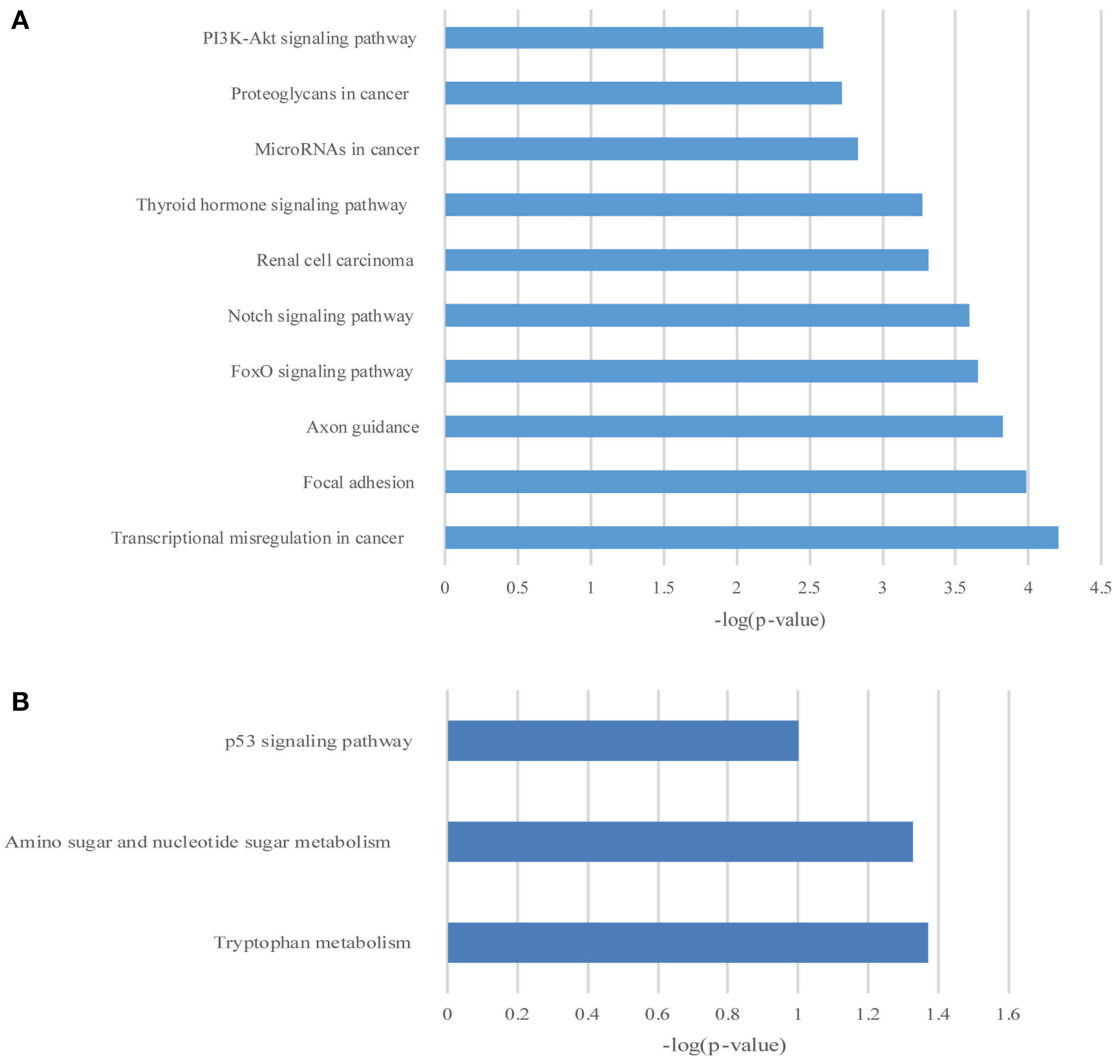
The KEGG pathway enrichment analysis on differentially expressed mRNAs. The top 10 enrichment scores in the KEGG pathway analysis of the up-regulated mRNAs **(A)** and top 3 down-regulated mRNAs **(B)**.

GO and KEGG enrichment analysis were also performed with 990 differentially expressed mRNAs. Finally, in the analysis of up-regulated mRNAs, 84, 58, and 34 GO terms were significantly enriched in the biological process, cell component and molecular function, respectively (Figure 6A). In the analysis of down-regulated mRNAs, 33, 7, 15 GO terms were significantly enriched in the biological process, cell component and molecular function, respectively (Figure 6B). The top 10 results of GO analysis on mRNA are shown in Figure 5. KEGG analysis showed that 29 pathways were involved in the mRNA downregulation caused by propofol (top 10 are shown in Figure 7A), and only 2 pathways related to propofol-regulated mRNAs were significantly enriched (top 3 are shown in Figure 7B). Among these significantly enriched pathways, MAPK and FoxO signaling pathways also appeared in the top 10 results of the KEGG analysis of lncRNA target genes, indicating the potential connection between these pathways and the effects of propofol (Figure 7).

### Construction of the co-expression network

**Figure 8:**
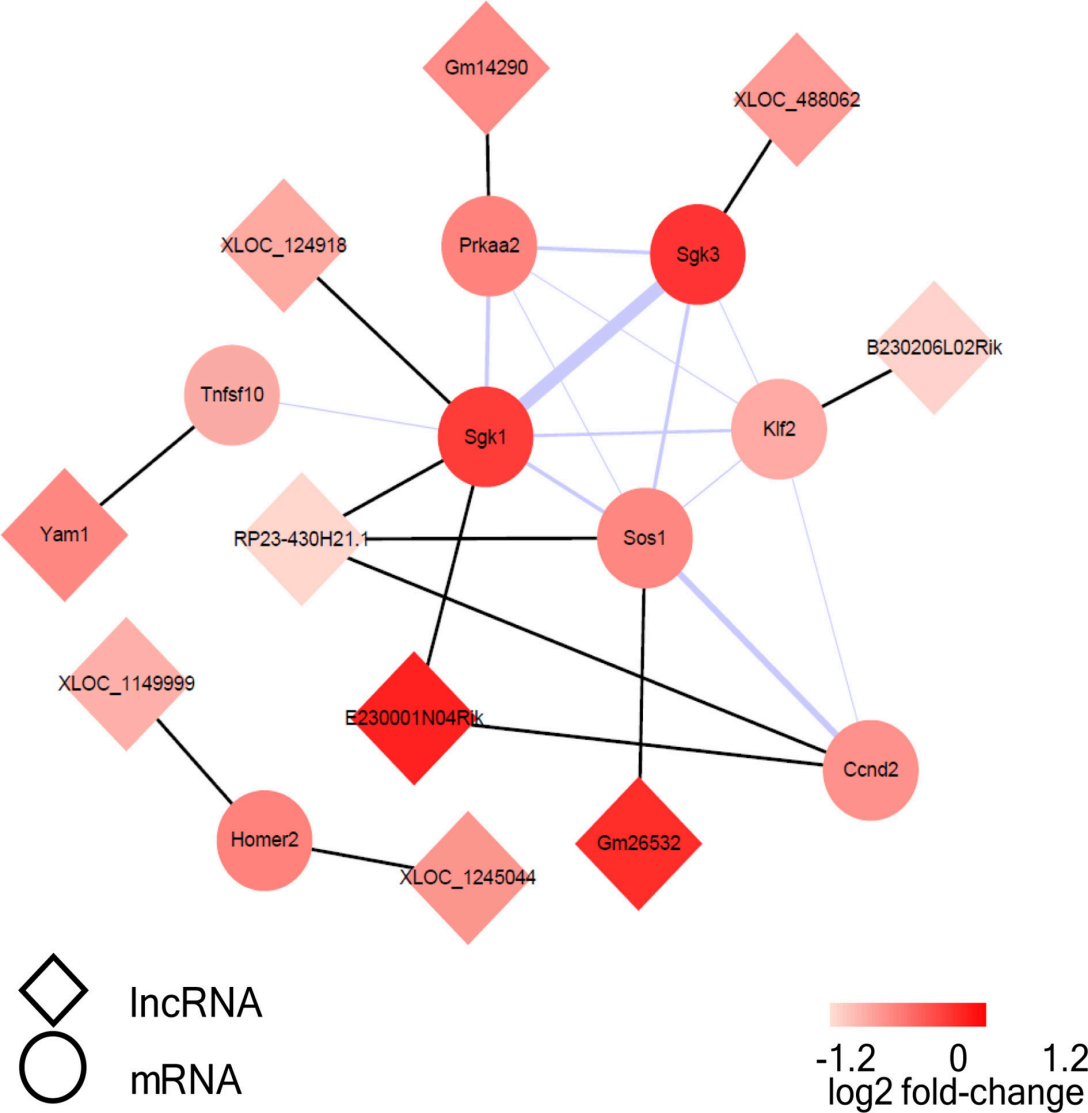
The visualization of the lncRNA/FoxO gene co-expression network in FoxO pathways. The circular nodes represent the FoxO-genes, and the diamond nodes represent the FoxO-lncRNAs. The black lines show connections between lncRNAs and their target mRNAs; the purple line shows integration of protein-protein interactions and the thicker lines represent the larger combined score.

A co-expression network of the dysregulated lncRNAs and their target mRNAs, which were involved in FoxO signaling pathway, was constructed. The co-expression network was composed of 13 lncRNA-mRNA predicted interactions and 13 protein-protein interactions. Sgk1, Sgk3, and Sos1 were identified as the hub nodes in the network (Figure 8). Moreover, lncRNA RP23-430H21.1 had three targets (Sgk1, Ccnd2, Sos1) and E230001N04Rik had two target mRNAs (Sgk1, Ccnd2), while the others had only one target in the network.

### Validation of the selected RNAs and proteins

**Figure 9:**
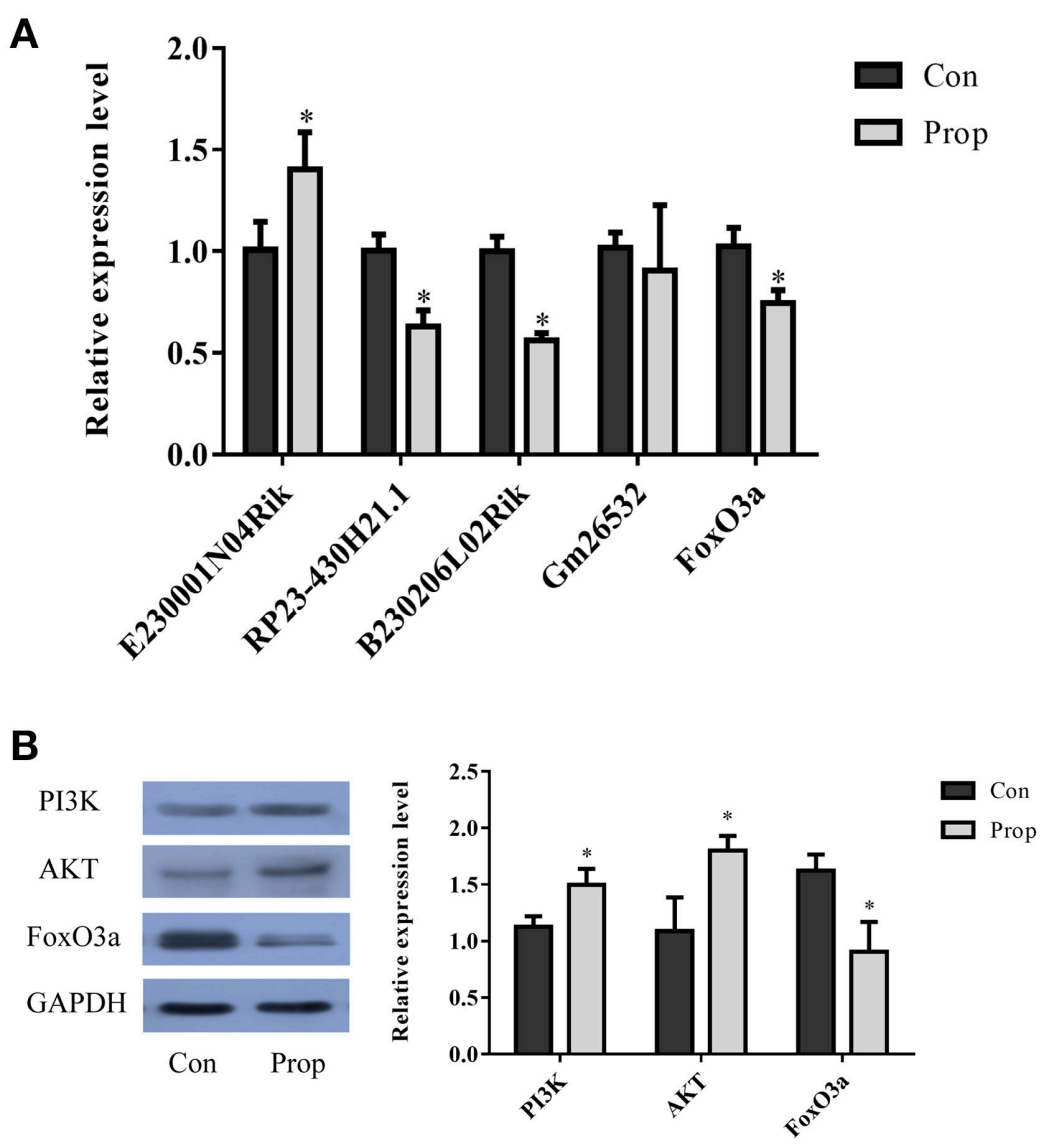
Validation of selected lncRNAs and proteins in the FoxO pathway**. (A)** Three of the qRT-PCR-validated lncRNAs (E230001N04Rik, RP23-430H21.1, B230206L02Rik and Gm26532) showed the same fold change patterns as those in the RNA-Seq results. The differences in Gm26532 were not statistically significant. **(B)** Quantitative analysis of selected proteins in the FoxO pathway that are differentially expressed in the Prop group vs. Con group. The PI3K and AKT are significantly up-regulated, but FoxO3a is decreasing in the Prop group. Data was normalized to the house keeping gene U6 (lncRNA) or GAPDH (mRNA), ^*^P < 0.05.

With a threshold of |FC| >1, 4 lncRNAs (E230001N04Rik, RP23-430H21.1, B230206L02Rik and Gm26532) in the network were selected for further validation by qRT-PCR. The up-regulated lncRNA, E230001N04Rik (*P* = 0.039), and 2 down-regulated lncRNAs, RP23-430H21.1 (*P* = 0.004) and B230206L02Rik (*P* = 0.001), showed the same fold change patterns as those in the RNA-Seq results, while down-regulated lncRNA Gm26532 (*P* = 0.585) did not reach statistical significance (Figure 9A). Quantitative analysis of FoxO pathway relative molecules showed that FoxO3a was down-regulated and PI3K/AKT were up-regulated in the Prop group (Figures 9A,B).

## Discussion

In this study, we found that 6 h of propofol sedation impaired spatial learning and memory abilities in mice, determined by the MWM test. A total of 146 differentially expressed lncRNA and 1103 mRNAs, including known transcripts and novel transcripts, were differentially expressed in the hippocampus, identified by RNA-Seq. Bioinformatic analysis, including GO analysis, pathway analysis, and network analysis suggested that the FoxO signaling pathway played an important role in the effect of propofol on the hippocampus. Four lncRNAs were selected from the FoxO-related network for further validation through qRT-PCR and 3 of them showed the same fold change patterns as those in the RNA-Seq results. Taken together, these results suggest that lncRNAs may play a complicated role in propofol-induced hippocampal dysfunction, which may contribute to the impairment of related spatial learning and memory functions.

The aim of this study was to identify the difference in the lncRNA and mRNA expression profiles in the hippocampus, in a propofol-induced neurotoxicity mice model. Propofol has been wildly used, in multiple clinical settings (Moseley et al., 1988), and its sedative effect may partly enhance the activity of GABA_A_R in the subsynaptic membrane (Li et al., 2015). Till date, the neuroprotective or neurotoxic effects of propofol on the CNS have been controversial, following multiple studies in different cerebral regions and various models (Velly et al., 2003; Erasso et al., 2013; Krzisch et al., 2013; Twaroski et al., 2015; Wang et al., 2015). In the present study, we adopted a propofol-induced neurotoxicity mice model which may impair adult neurogenesis in the hippocampus (Krzisch et al., 2013). Krzisch et al. (2013) found that propofol-induced anesthesia significantly decreased the survival and maturation of adult-born hippocampal neurons, in a developmental, stage-dependent manner. The results of our Morris water maze experiments demonstrated that propofol sedation increases the escape latency of MWM, and decreases the number of platform crossings during the probe test, which suggests that propofol exposure impairs spatial learning and memory in adult mice. The results from the study by Krzisch's group and those from our behavior test, are both in support of propofol-induced neurotoxicity. The mechanism of propofol-induced neurotoxicity has gained increasing attention over the last few decades. Recently published studies suggested that propofol-induced neurotoxicity may be regulated by microRNA. For instance, propofol may induce human embryonic stem cell-derived neuronal death through a signal transducer, and activation of the transcription 3/miR-21/ Sprouty 2-dependent mechanism (Twaroski et al., 2014). Rno-miR-665 is involved in the neurotoxicity induced by propofol via a caspase-3, through negative regulation of BCL2L1 (Sun et al., 2015). However, the role of lncRNA in propofol-related neurotoxicity remains unclear. Thus, it is meaningful to investigate the differences in lncRNA expression, in mice hippocampi, following propofol exposure.

LncRNAs, a class of non-coding RNA molecules, are no longer than 200 nucleotides in length and have barely discernable coding potential. They are widely involved in multiple pathophysiological processes (Guttman et al., 2009; Song et al., 2016; Wang et al., 2016), and also present a region-specific function (Derrien et al., 2012). LncRNAs in the CNS participate in many aspects of brain function and their role in CNS development range from early neural differentiation to late-stage synaptogenesis (Briggs et al., 2015). Research indicates that lncRNAs play an intricate part in NSCs, neuronal proliferation and apoptosis (Ramos et al., 2015; Lv, 2017). In our study, we performed RNA-Seq to screen the differential expression of lncRNA and mRNA in the hippocampus, after propofol sedation. LncRNA Malat1, one of the down-regulated lncRNAs following propofol sedation, was a highly conserved lncRNA that has been found in various cancers, such as non-small cell lung cancer (Ji et al., 2003) and breast cancer (Feng et al., 2016), and plays a role in the proliferation of myocardial cells (Zhao et al., 2015). More importantly, Bernard (Bernard et al., 2010) also discovered that Malat1 can regulate the synaptic plasticity of primary culture neurons. The results of our RNA-Seq suggested that propofol can reduce the expression of Malat1. LncRNA Kcnq1ot1 has been known to be associated with Beckwith-Wiedemann Syndrome and glioma malignancy (Gong et al., 2017), and depressing lung adenocarcinoma chemoresistance to paclitaxel (Ren et al., 2017). In addition, our results revealed that LncRNA Kcnq1ot1 significantly increased following propofol sedation in mice hippocampi. Overall, we screened 146 differentially expressed lncRNAs in the hippocampus through RNA-Seq. For the first time, through our study we have presented an lncRNA expression profile in mice hippocampus following propofol exposure. These results may also indicate the role of lncRNA and the underlying molecular mechanism of propofol-related neurotoxicity.

To effectively screen the candidate lncRNAs for further validation and research, bioinformatics analysis was conducted, separately, for mRNAs and the target genes of lncRNAs. The results showed several important pathways that were significantly enriched, particularly the FoxO signaling pathway, which was significantly enriched in both mRNAs and the target genes and indicated that this pathway may play a critical role in the dysfunction of the hippocampus, after propofol exposure. The FoxO family belongs to an evolutionarily conserved group of forkhead transcription factors. Mammals have four isoforms of the FoxO family: FoxO 1 (FKHR), 3a (FKHRL1), 4 (AFX), and FoxO6 (Hwangbo et al., 2004). FoxO transcription factors are at the interface of diverse physiological processes, e.g., coordinating gene expression that regulates proliferation, cell-cycle, DNA repair, oxidative stress resistance as well as metabolism (Medema et al., 2000; Nakae et al., 2001). Nutrient and energy stress signaling pathways regulate FoxOs and are important for NSC maintenance (Rafalski and Brunet, 2011; Spéder et al., 2011). In the adult mouse brain, FoxO1 is most abundant in the striatum, dentate gyrus, and the ventral hippocampus (Paik et al., 2009), while FoxO3a is highly expressed in the cortex, hippocampus, and cerebellum (Hoekman et al., 2006). Previous studies have shown that the PI3K/Akt-FoxO signaling pathway plays a central role in the development of the nervous system. Lisa (Kennedy et al., 2013) demonstrated that the insulin/IGF-1-PI3K signaling pathway modulates the activity of the DAF-16/FoxO transcription factor to promote the anterior migrations of the hermaphrodite-specific neurons during embryogenesis of *C. elegans* (by signaling pathways that are conserved in humans). FoxO also restricts growth and survival of apoptosis-inhibited mushroom body neuroblasts, while activation of the growth-promoting insulin/PI3 kinase pathway sustains not only long-term survival of adult mushroom body neuroblasts, but also increases their proliferation and growth rate (Paik et al., 2009). However, lncRNA-mediated regulation of the FoxO signaling pathway in nervous system development is yet to be elucidated. To clarify the relationship between lncRNAs and FoxO signaling pathway, we constructed a protein-protein interaction network and validated lncRNAs (E230001N04Rik, RP23-430H21.1, B230206L02Rik, and Gm26532) and FoxO3a mRNA via qRT-PCR. Furthermore, western blotting was performed to evaluate the PI3K/AKT/FoxO3a protein. According to our bioinformatic analysis and western blotting results, we hypothesize that these lncRNAs, E230001N04Rik, RP23-430H21.1, and B230206L02Rik, may participate in the FoxO signaling pathway, to regulate propofol-induced neurotoxicity. Nevertheless, the detailed regulatory mechanism of these lncRNAs in the FoxO signaling pathway is still require further study in order to confirm these findings.

In conclusion, our study reveals that propofol sedation can influence the expression of lncRNAs and mRNAs in the hippocampus, and bioinformatics analysis have identified several key biological processes and KEGG pathways associated with propofol sedation. Our results provide a framework for further study on the role of lncRNAs in propofol-induced or -related neurotoxicity, particularly with regards to hippocampus-related dysfunction.

## Author contributions

JF and QZ: Carried out most procedures of this study, participated in the data analysis, and manuscript writing; YL and XS: Participated in design of animal study and interpreted part data of this study; JT, ZQ, and JH: Participated in the collection of data, data analysis; TT, ZQ, and JT: Designed this study, provided financial support, and wrote the manuscript. All authors read and approved the final manuscript.

### Conflict of interest statement

The authors declare that the research was conducted in the absence of any commercial or financial relationships that could be construed as a potential conflict of interest.

## References

[B1] Bernard D. Prasanth K. V. Tripathi V. Colasse S. Nakamura T. Xuan Z.. (2010). A long nuclear-retained non-coding RNA regulates synaptogenesis by modulating gene expression. EMBO J. 29, 3082–3093. 10.1038/emboj.2010.199 20729808 PMC2944070

[B2] Briggs J. A. Wolvetang E. J. Mattick J. S. Rinn J. L. Barry G. (2015). Mechanisms of long Non-coding RNAs in mammalian nervous system development, plasticity, disease, and evolution. Neuron 88, 861–877. 10.1016/j.neuron.2015.09.045 26637795

[B3] Bunch H. (2018). Gene regulation of mammalian long non-coding RNA. Mol. Genet. Genomics 293, 1–15. 10.1007/s00438-017-1370-9 28894972

[B4] Cabili M. N. Trapnell C. Goff L. Koziol M. Tazon-Vega B. Regev A.. (2011). Integrative annotation of human large intergenic noncoding RNAs reveals global properties and specific subclasses. Genes Dev. 25, 1915–1927. 10.1101/gad.17446611 21890647 PMC3185964

[B5] Chen B. Deng X. Wang B. Liu H. (2016). Etanercept, an inhibitor of TNF-a, prevents propofol-induced neurotoxicity in the developing brain. Int. J. Dev. Neurosci. 55, 91–100. 10.1016/j.ijdevneu.2016.10.002 27756568

[B6] Deng X. Chen B. Wang B. Zhang J. Liu H. (2017). TNF-alpha mediates the intrinsic and extrinsic pathway in Propofol-Induced neuronal apoptosis via PI3K/Akt signaling pathway in rat prefrontal cortical neurons. Neurotox. Res. 32, 409–419. 10.1007/s12640-017-9751-8 28540664

[B7] Derrien T. Johnson R. Bussotti G. Tanzer A. Djebali S. Tilgner H.. (2012). The GENCODE v7 catalog of human long noncoding RNAs: analysis of their gene structure, evolution, and expression. Genome Res. 22, 1775–1789. 10.1101/gr.132159.111 22955988 PMC3431493

[B8] Erasso D. M. Camporesi E. M. Mangar D. Saporta S. (2013). Effects of isoflurane or propofol on postnatal hippocampal neurogenesis in young and aged rats. Brain Res. 1530, 1–12. 10.1016/j.brainres.2013.07.035 23891717

[B9] Fan J. Zhou Q. Qin Z. Tao T. (2016). Effect of propofol on microRNA expression in rat primary embryonic neural stem cells. BMC Anesthesiol. 16:95. 10.1186/s12871-016-0259-1 27737635 PMC5064799

[B10] Feng T. Shao F. Wu Q. Zhang X. Xu D. Qian K.. (2016). miR-124 downregulation leads to breast cancer progression via LncRNA-MALAT1 regulation and CDK4/E2F1 signal activation. Oncotarget 7, 16205–16216. 10.18632/oncotarget.7578 26918449 PMC4941308

[B11] Gong W. Zheng J. Liu X. Liu Y. Guo J. Gao Y.. (2017). Knockdown of long Non-Coding RNA KCNQ1OT1 restrained glioma cells' malignancy by activating miR-370/CCNE2 axis. Front. Cell. Neurosci. 11:84. 10.3389/fncel.2017.00084 28381990 PMC5360732

[B12] Guttman M. Amit I. Garber M. French C. Lin M. F. Feldser D.. (2009). Chromatin signature reveals over a thousand highly conserved large non-coding RNAs in mammals. Nature 458, 223–227. 10.1038/nature07672 19182780 PMC2754849

[B13] Guttman M. Garber M. Levin J. Z. Donaghey J. Robinson J. Adiconis X.. (2010). Ab initio reconstruction of cell type-specific transcriptomes in mouse reveals the conserved multi-exonic structure of lincRNAs. Nat. Biotechnol. 28, 503–U166. 10.1038/nbt.1633 20436462 PMC2868100

[B14] Hoekman M. F. Jacobs F. M. Smidt M. P. Burbach J. P. (2006). Spatial and temporal expression of FoxO transcription factors in the developing and adult murine brain. Gene Expr. Patterns 6, 134–140. 10.1016/j.modgep.2005.07.003 16326148

[B15] Hsiao H. T. Wu H. Huang P. C. Tsai Y. C. Liu Y. C. (2016). The effect of propofol and sevoflurane on antioxidants and proinflammatory cytokines in a porcine ischemia-reperfusion model. Acta Anaesthesiol. Taiwan 54, 6–10. 10.1016/j.aat.2015.11.002 26688227

[B16] Hwangbo D. S. Gersham B. Tu M. P. Palmer M. Tatar M. (2004). Drosophila dFOXO controls lifespan and regulates insulin signalling in brain and fat body. Nature 429, 562–566. 10.1038/nature02549 15175753

[B17] Jandura A. Krause H. M. (2017). The new RNA world: growing evidence for long noncoding rna functionality. Trends Genet. 33, 665–676. 10.1016/j.tig.2017.08.002 28870653

[B18] Ji P. Diederichs S. Wang W. Böing S. Metzger R. Schneider P. M.. (2003). MALAT-1, a novel noncoding RNA, and thymosin beta4 predict metastasis and survival in early-stage non-small cell lung cancer. Oncogene 22, 8031–8041. 10.1038/sj.onc.1206928 12970751

[B19] Jiang Q. Wang Y. Shi X. (2017). Propofol inhibits neurogenesis of rat neural stem cells by upregulating MicroRNA-141-3p. Stem Cells Dev. 26, 189–196. 10.1089/scd.2016.0257 27796156

[B20] Kennedy L. M. Pham S. C. Grishok A. (2013). Nonautonomous regulation of neuronal migration by insulin signaling, DAF-16/FOXO, and PAK-1. Cell Rep. 4, 996–1009. 10.1016/j.celrep.2013.07.045 23994474 PMC3800683

[B21] Kong L. Zhang Y. Ye Z. Q. Liu X. Q. Zhao S. Q. Wei L.. (2007). CPC: assess the protein-coding potential of transcripts using sequence features and support vector machine. Nucleic Acids Res. 35, W345–W349. 10.1093/nar/gkm391 17631615 PMC1933232

[B22] Krzisch M. Sultan S. Sandell J. Demeter K. Vutskits L. Toni N. (2013). Propofol anesthesia impairs the maturation and survival of adult-born hippocampal neurons. Anesthesiology 118, 602–610. 10.1097/ALN.0b013e3182815948 23314165

[B23] Langfelder P. Horvath S. (2008). WGCNA: an R package for weighted correlation network analysis. BMC Bioinformatics 9:599. 10.1186/1471-2105-9-559 19114008 PMC2631488

[B24] Li Y. Wu Y. Li R. Wang C. Jia N. Zhao C.. (2015). Propofol regulates the surface expression of GABAA receptors: implications in synaptic inhibition. Anesth. Analg. 121, 1176–1183. 10.1213/ANE.0000000000000884 26241086

[B25] Lin M. F. Jungreis I. Kellis M. (2011). PhyloCSF: a comparative genomics method to distinguish protein coding and non-coding regions. Bioinformatics 27, I275–I282. 10.1093/bioinformatics/btr209 21685081 PMC3117341

[B26] Liu Y. Yan Y. Inagaki Y. Logan S. Bosnjak Z. J. Bai X. (2017). Insufficient Astrocyte-Derived Brain-Derived neurotrophic factor contributes to Propofol-Induced neuron death through Akt/Glycogen synthase kinase 3beta/Mitochondrial fission pathway. Anesth. Analg. 125, 241–254. 10.1213/ANE.0000000000002137 28622174 PMC5484590

[B27] Lv H. R. (2017). lncRNA-Map2k4 sequesters miR-199a to promote FGF1 expression and spinal cord neuron growth. Biochem. Biophys. Res. Commun. 490, 948–954. 10.1016/j.bbrc.2017.06.145 28655615

[B28] Medema R. H. Kops G. J. Bos J. L. Burgering B. M. (2000). AFX-like Forkhead transcription factors mediate cell-cycle regulation by Ras and PKB through p27kip1. Nature 404, 782–787. 10.1038/35008115 10783894

[B29] Moseley H. Shankar K. B. Kumar Y. Hallsworth R. Krishnan A. (1988). Propofol: a new intravenous anaesthetic. West Indian Med. J. 37, 229–231. 3266048

[B30] Nakae J. Kitamura T. Silver D. L. Accili D. (2001). The forkhead transcription factor FoxO1 (Fkhr) confers insulin sensitivity onto glucose-6-phosphatase expression. J. Clin. Invest. 108, 1359–1367. 10.1172/JCI200112876 11696581 PMC209440

[B31] Nie Y. Lu Y. X. Lv L. H. (2015). Effect of propofol on generation of inflammatory mediator of monocytes. Asian Pac. J. Trop. Med. 8, 964–970. 10.1016/j.apjtm.2015.10.008 26614998

[B32] Paik J. H. Ding Z. Narurkar R. Ramkissoon S. Muller F. Kamoun W. S.. (2009). FoxOs cooperatively regulate diverse pathways governing neural stem cell homeostasis. Cell Stem Cell 5, 540–553. 10.1016/j.stem.2009.09.013 19896444 PMC3285492

[B33] Punta M. Coggill P. C. Eberhardt R. Y. Mistry J. Tate J. Boursnell C.. (2012). The Pfam protein families database. Nucleic Acids Res. 40, D290–D301. 10.1093/nar/gkr1065 22127870 PMC3245129

[B34] Qiao H. Li Y. Xu Z. Li W. Fu Z. Wang Y.. (2017). Propofol affects neurodegeneration and neurogenesis by regulation of autophagy via effects on intracellular calcium homeostasis. Anesthesiology 127, 490–501. 10.1097/ALN.0000000000001730 28614084 PMC5561483

[B35] Rafalski V. A. Brunet A. (2011). Energy metabolism in adult neural stem cell fate. Prog. Neurobiol. 93, 182–203. 10.1016/j.pneurobio.2010.10.007 21056618

[B36] Ramos A. D. Andersen R. E. Liu S. J. Nowakowski T. J. Hong S. J. Gertz C.. (2015). The long noncoding RNA Pnky regulates neuronal differentiation of embryonic and postnatal neural stem cells. Cell Stem Cell 16, 439–447. 10.1016/j.stem.2015.02.007 25800779 PMC4388801

[B37] Ren K. Xu R. Huang J. Zhao J. Shi W. (2017). Knockdown of long non-coding RNA KCNQ1OT1 depressed chemoresistance to paclitaxel in lung adenocarcinoma. Cancer Chemother. Pharmacol. 80, 243–250. 10.1007/s00280-017-3356-z 28600629

[B38] Rinn J. L. Chang H. Y. (2012). Genome regulation by long noncoding RNAs. Annu. Rev. Biochem. 81, 145–166. 10.1146/annurev-biochem-051410-092902 22663078 PMC3858397

[B39] Samir A. Gandreti N. Madhere M. Khan A. Brown M. Loomba V. (2015). Anti-inflammatory effects of propofol during cardiopulmonary bypass: a pilot study. Ann. Card. Anaesth. 18, 495–501. 10.4103/0971-9784.166451 26440235 PMC4881689

[B40] Song C. Zhang J. Liu Y. Pan H. Qi H. P. Cao Y. G.. (2016). Construction and analysis of cardiac hypertrophy-associated lncRNA-mRNA network based on competitive endogenous RNA reveal functional lncRNAs in cardiac hypertrophy. Oncotarget 7, 10827–10840. 10.18632/oncotarget.7312 26872060 PMC4905442

[B41] Spéder P. Liu J. Brand A. H. (2011). Nutrient control of neural stem cells. Curr. Opin. Cell Biol. 23, 724–729. 10.1016/j.ceb.2011.08.004 21930368

[B42] Sun L. Luo H. Bu D. Zhao G. Yu K. Zhang C.. (2013). Utilizing sequence intrinsic composition to classify protein-coding and long non-coding transcripts. Nucleic Acids Res. 41:e166. 10.1093/nar/gkt646 23892401 PMC3783192

[B43] Sun W. C. Liang Z. D. Pei L. (2015). Propofol-induced rno-miR-665 targets BCL2L1 and influences apoptosis in rodent developing hippocampal astrocytes. Neurotoxicology 51, 87–95. 10.1016/j.neuro.2015.08.001 26254736

[B44] Sun W. Yang Y. Xu C. Guo J. (2017). Regulatory mechanisms of long noncoding RNAs on gene expression in cancers. Cancer Genet. 216–217, 105–110. 10.1016/j.cancergen.2017.06.003 29025584

[B45] Szklarczyk D. Morris J. H. Cook H. Kuhn M. Wyder S. Simonovic M.. (2017). The STRING database in 2017: quality-controlled protein-protein association networks, made broadly accessible. Nucleic Acids Res. 45, D362–D368. 10.1093/nar/gkw937 27924014 PMC5210637

[B46] Tan M. C. Widagdo J. Chau Y. Q. Zhu T. Wong J. J. Cheung A.. (2017). The Activity-Induced long Non-Coding RNA Meg3 modulates AMPA receptor surface expression in primary cortical neurons. Front. Cell. Neurosci. 11:124. 10.3389/fncel.2017.00124 28515681 PMC5413565

[B47] Trapnell C. Roberts A. Goff L. Pertea G. Kim D. Kelley D. R.. (2012). Differential gene and transcript expression analysis of RNA-seq experiments with TopHat and Cufflinks. Nat. Protoc. 7, 562–578. 10.1038/nprot.2012.016 22383036 PMC3334321

[B48] Twaroski D. M. Yan Y. Olson J. M. Bosnjak Z. J. Bai X. (2014). Down-regulation of MicroRNA-21 is involved in the Propofol-induced neurotoxicity observed in human stem Cell-derived neurons. Anesthesiology 121, 786–800. 10.1097/ALN.0000000000000345 24950164 PMC4174986

[B49] Twaroski D. M. Yan Y. Zaja I. Clark E. Bosnjak Z. J. Bai X. (2015). Altered mitochondrial dynamics contributes to Propofol-induced cell death in human stem Cell-derived neurons. Anesthesiology 123, 1067–1083. 10.1097/ALN.0000000000000857 26352374 PMC4632973

[B50] Ucar M. Ozgül U. Polat A. Toprak H. I. Erdogan M. A. Aydogan M. S.. (2015). Comparison of antioxidant effects of isoflurane and propofol in patients undergoing donor hepatectomy. Transplant. Proc. 47, 469–472. 10.1016/j.transproceed.2014.11.043 25769593

[B51] Velly L. J. Guillet B. A. Masmejean F. M. Nieoullon A. L. Bruder N. J. Gouin F. M.. (2003). Neuroprotective effects of propofol in a model of ischemic cortical cell cultures: role of glutamate and its transporters. Anesthesiology 99, 368–375. 10.1097/00000542-200308000-00018 12883409

[B52] Vutskits L. Gascon E. Tassonyi E. Kiss J. Z. (2005). Clinically relevant concentrations of propofol but not midazolam alter *in vitro* dendritic development of isolated gamma-aminobutyric acid-positive interneurons. Anesthesiology 102, 970–976. 10.1097/00000542-200505000-00016 15851884

[B53] Wang J. Ma R. Ma W. Chen J. Yang J. Xi Y.. (2016). LncDisease: a sequence based bioinformatics tool for predicting lncRNA-disease associations. Nucleic Acids Res. 44:e90. 10.1093/nar/gkw093 26887819 PMC4872090

[B54] Wang W. Lu R. Feng D. Y. Liang L. R. Liu B. Zhang H. (2015). Inhibition of microglial activation contributes to propofol-induced protection against post-cardiac arrest brain injury in rats. J. Neurochem. 134, 892–903. 10.1111/jnc.13179 26016627

[B55] Yan Y. Qiao S. Kikuchi C. Zaja I. Logan S. Jiang C.. (2017). Propofol induces apoptosis of neurons but not astrocytes, oligodendrocytes, or neural stem cells in the neonatal mouse hippocampus. Brain Sci. 7:130. 10.3390/brainsci7100130 29036908 PMC5664057

[B56] Zhang D. Zhou X. H. Zhang J. Zhou Y. X. Ying J. Wu G. Q.. (2015). Propofol promotes cell apoptosis via inhibiting HOTAIR mediated mTOR pathway in cervical cancer. Biochem. Biophys. Res. Commun. 468, 561–567. 10.1016/j.bbrc.2015.10.129 26523512

[B57] Zhao J. Li L. Peng L. (2015). MAPK1 up-regulates the expression of MALAT1 to promote the proliferation of cardiomyocytes through PI3K/AKT signaling pathway. Int. J. Clin. Exp. Pathol. 8, 15947–15953. 26884868 PMC4730081

[B58] Zhong L. Luo F. Zhao W. Feng Y. Wu L. Lin J.. (2016). Propofol exposure during late stages of pregnancy impairs learning and memory in rat offspring via the BDNF-TrkB signalling pathway. J. Cell. Mol. Med. 20, 1920–1931. 10.1111/jcmm.12884 27297627 PMC5020635

