# Supplementary material for: Profiling of Long Non-coding RNAs and mRNAs by RNA-Sequencing in the Hippocampi of Adult Mice Following Propofol Sedation
Source: Front Mol Neurosci. 2018 Mar 23;11:91. doi: 10.3389/fnmol.2018.00091 (PMC5876304; doi:10.3389/fnmol.2018.00091)
Supplement: Supplementary file 1 [file Table1.DOCX]

*Supplementary Material*

**Identification of long non-coding RNAs in hippocampus of adult mice after propofol sedation by RNA-sequencing**

Jun Fan^1#^, Quan Zhou^1#,^ Yan Li^1^, Xiuling Song^1^, Jijie Hu^2^, Jing Tang^1^, Zaisheng Qin^1^, Tao Tao^1*^

*Correspondence: M.D. & Ph.D Tao Tao, taotaomzk@smu.edu.cn

Supplementary Tables:

**Table 1**. Summary of sequence reads mapping to genome

| Sample name | Con 1 | Con 2 | Con 3 | Prop 1 | Prop 2 | Prop 3 |
| --- | --- | --- | --- | --- | --- | --- |
| Raw reads | 110835434 | 113637318 | 159614600 | 133756736 | 158979510 | 124294760 |
| Clean reads | 104346344 | 107298022 | 153814828 | 125722588 | 154268330 | 116936142 |
| Total mapped | 90829286 (87.05%) | 93972426 (87.58%) | 134514031 (87.45%) | 108863549 (86.59%) | 134903050 (87.45%) | 101952343 (87.19%) |
| Multiple mapped | 15974853 (15.31%) | 13409783 (12.5%) | 19651675 (12.78%) | 18437329 (14.67%) | 30769836 (19.95%) | 15715598 (13.44%) |
| Uniquely mapped | 74854433 (71.74%) | 80562643 (75.08%) | 114862356 (74.68%) | 90426220 (71.93%) | 104133214 (67.5%) | 86236745 (73.75%) |
| Read-1 | 39268858 (37.63%) | 42201635 (39.33%) | 59971900 (38.99%) | 47662804 (37.91%) | 52751953 (34.19%) | 45384437 (38.81%) |
| Read-2 | 35585575 (34.1%) | 38361008 (35.75%) | 54890456 (35.69%) | 42763416 (34.01%) | 51381261 (33.31%) | 40852308 (34.94%) |
| Reads map to '+' | 37499080 (35.94%) | 40347433 (37.6%) | 57518744 (37.39%) | 45306649 (36.04%) | 52120063 (33.79%) | 43198101 (36.94%) |
| Reads map to '-' | 37355353 (35.8%) | 40215210 (37.48%) | 57343612 (37.28%) | 45119571 (35.89%) | 52013151 (33.72%) | 43038644 (36.81%) |
| Non-splice reads | 54103684 (51.85%) | 59720992 (55.66%) | 85298889 (55.46%) | 65254839 (51.9%) | 74418792 (48.24%) | 62438605 (53.4%) |
| Splice reads | 20750749 (19.89%) | 20841651 (19.42%) | 29563467 (19.22%) | 25171381 (20.02%) | 29714422 (19.26%) | 23798140 (20.35%) |
