# Supplementary material for: Profiling of Long Non-coding RNAs and mRNAs by RNA-Sequencing in the Hippocampi of Adult Mice Following Propofol Sedation
Source: Front Mol Neurosci. 2018 Mar 23;11:91. doi: 10.3389/fnmol.2018.00091 (PMC5876304; doi:10.3389/fnmol.2018.00091)
Supplement: Supplementary file 2 [file Table2.DOCX]

*Supplementary Material*

**Identification of long non-coding RNAs in hippocampus of adult mice after propofol sedation by RNA-sequencing**

Jun Fan^1#^, Quan Zhou^1#,^ Yan Li^1^, Xiuling Song^1^, Jijie Hu^2^, Jing Tang^1^, Zaisheng Qin^1^, Tao Tao^1*^

*Correspondence: M.D. & Ph.D Tao Tao, taotaomzk@smu.edu.cn

Supplementary Tables:

| Samples | Con 1 | Con 2 | Con 3 | Prop 1 | Prop 2 | Prop 3 |
| --- | --- | --- | --- | --- | --- | --- |
| protein_coding | 32428288 (83.69%) | 35317349 (84.84%) | 48983212 (82.89%) | 38488324 (81.92%) | 43207891 (82.53%) | 37147882 (83.18%) |
| lincRNA | 529990 (1.37%) | 605344 (1.45%) | 899896 (1.52%) | 700339 (1.49%) | 626642 (1.20%) | 722136 (1.62%) |
| miRNA | 2593 (0.01%) | 2355 (0.01%) | 3407 (0.01%) | 2394 (0.01%) | 4263 (0.01%) | 2759 (0.01%) |
| Mt_rRNA | 250328 (0.65%) | 230076 (0.55%) | 300556 (0.51%) | 255955 (0.54%) | 462283 (0.88%) | 235079 (0.53%) |
| Mt_tRNA | 1071 (0.00%) | 990 (0.00%) | 1479 (0.00%) | 1742 (0.00%) | 2089 (0.00%) | 1225 (0.00%) |
| antisense | 62731 (0.16%) | 64833 (0.16%) | 105331 (0.18%) | 86626 (0.18%) | 78845 (0.15%) | 77989 (0.17%) |
| misc_RNA | 1501964 (3.88%) | 1339712 (3.22%) | 1956645 (3.31%) | 1782162 (3.79%) | 3165975 (6.05%) | 1535586 (3.44%) |
| processed_transcript | 149979 (0.39%) | 173630 (0.42%) | 254493 (0.43%) | 190151 (0.40%) | 199726 (0.38%) | 182409 (0.41%) |
| pseudogene | 74556 (0.19%) | 77095 (0.19%) | 99973 (0.17%) | 86492 (0.18%) | 90480 (0.17%) | 81187 (0.18%) |
| sense_intronic | 881 (0.00%) | 1312 (0.00%) | 1602 (0.00%) | 1106 (0.00%) | 981 (0.00%) | 868 (0.00%) |
| sense_overlapping | 4779 (0.01%) | 5787 (0.01%) | 8190 (0.01%) | 5511 (0.01%) | 5476 (0.01%) | 5448 (0.01%) |
| snRNA | 1039 (0.00%) | 802 (0.00%) | 1421 (0.00%) | 1333 (0.00%) | 1959 (0.00%) | 1288 (0.00%) |
| snoRNA | 12005 (0.03%) | 9407 (0.02%) | 16714 (0.03%) | 14642 (0.03%) | 21582 (0.04%) | 12186 (0.03%) |
| Others | 3728797 (9.62%) | 3798849 (9.13%) | 6457171 (10.93%) | 5364144 (11.42%) | 4482461 (8.56%) | 4650556 (10.41%) |

**Table 2**. The distribution of sequence reads mapping to the known gene types
