# Supplementary material for: Profiling of Long Non-coding RNAs and mRNAs by RNA-Sequencing in the Hippocampi of Adult Mice Following Propofol Sedation
Source: Front Mol Neurosci. 2018 Mar 23;11:91. doi: 10.3389/fnmol.2018.00091 (PMC5876304; doi:10.3389/fnmol.2018.00091)
Supplement: Supplementary file 3 [file Table3.docx]

Table S3. The primer of selected lncRNAs and mRNAs

| lncRNA name | Forward primer (5'-3') | Reverse primer (5'-3') |
| --- | --- | --- |
| E230001N04Rik | GGAAGCAAGCCAGTAAATAG | ATCACCAAAGGAAGTCAGG |
| RP23-430H21.1 | TCTTCCAGTCGGTGTTTG | TGCCTTCGTTTTGCTATT |
| B230206L02Rik | ATGGAAAGCAGGAAGGAG | TTATTGGTGTGGGTGTGG |
| Gm26532 | AGACCTGGAAACAATGGG | ATCTATCTGCTTTGGGGAA |
| U6 | CTCGCTTCGGCAGCACA | AACGCTTCACGAATTTGCGT |
| FoxO3a | ACCGTGTACCGTGGAGTTGC | GCTGGGAGGAAGGGAGTGTT |
| GAPDH | GGGTCCCAGCTTAGGTTCATC | CGGGACGAGGAAACACTCTC |
